# Deformation Mechanism, Microstructure, and Mechanical Properties Evolution of Mg–Gd–Y–Zr Alloy during Cold Torsion

**DOI:** 10.3390/ma14082067

**Published:** 2021-04-20

**Authors:** Hongchao Xiao, Zhengjiang Yang, Jie Li, Yingchun Wan

**Affiliations:** 1 School of Metallurgy and Environment, Central South University, Changsha 410083, China; hcxiao@csu.edu.cn (H.X.); jielijie@csu.edu.cn (J.L.); 2 School of Materials Science and Engineering, Central South University, Changsha 410083, China; zjyangmse@csu.edu.cn; 3 Light Alloy Research Institute, Central South University, Changsha 410083, China

**Keywords:** torsion, Mg–Gd–Y–Zr, deformation mechanism, microstructure

## Abstract

Mg–Gd–Y–Zr alloy was subjected to torsion of various strain levels at room temperature. Obvious traces of basal slip were observed in the twisted alloy. Dislocations of <c+a> were also observed, but there were no signs of significant sliding. Even in the sample whose equivalent strain became 0.294, $\left(10\bar{1}0\right)$ twinning and $\left(10\bar{1}2\right)$ twinning were rarely seen. The deformation mode with predominant basal <a> dislocations and subordinate <c+a> dislocations resulted in a modified Y fiber texture with a basal pole slightly dispersed at about 70° from the twist axis. Mechanical tests revealed that the tensile strength and compressive strengths increased simultaneously after twisting.

## 1. Introduction

Due to high specific strength, high specific stiffness, and high damping capacity, from the perspective of weight reduction, magnesium alloys are becoming the most promising candidates for replacing aluminum alloys and steels in the fields of automobiles, rail transit, aerospace and defense, energy saving, emission control, and vibration absorption. In recent years, significant efforts have been made to overcome the shortcomings of magnesium alloys, such as low strength and low ductility, which limit their application in engineering practice [1,2,3]. Various reports have revealed that Mg alloys generally exhibit an excellent strengthening response to plastic deformation due to dynamic recrystallization (DRX)-induced grain refinement [4,5], twinning-induced [6], or micro-shear band-induced [7] grain breakage and division. The cold deformation below the DRX temperature has been proven to effectively adjust the microstructure and texture to obtain the desired mechanical properties [8,9,10]. For example, cold rolling on fine-grained ZK60 alloy introduces a large number of nano-scale deformation bands, which helps to enhance dislocation accumulation and strain hardening [11]. The cold strain during the drawing process of AZ31 alloy results in appreciable twinning and micro-shear bands, which helps to further break the grains into nano-grains [12]. The recrystallized grains obtained in the cold-rolled AZ31 alloy have random orientations and weaken the structure in the early stage of the deformation [13].

Torsional deformation can provide uniform deformation in the longitudinal direction and produce gradient strain in the radial direction. It has been observed that torsion at room temperature can enhance the comprehensive mechanical properties of magnesium alloys. For example, the torsional deformation of AZ31 alloy can help strengthen the alloy by generating twin lamellae, profile dislocations, and weakened texture [14], while the pre-twinned AZ31 alloy shows higher strength [15]. It has also been observed that torsion can improve the tensile strength, compressive strength, and asymmetry of AZ91 alloy [16], and the strength of the alloy increases with the twisting angle.

Recently, it has frequently been reported that the addition of Gd and Y to magnesium alloys is notably effective in alloy enhancement because of the remarkable aging response caused by precipitation [17,18], and the asymmetry reduction by weakened texture [19]. Mg–Gd–Y alloys have significant advantages in developing wrought magnesium alloys with excellent mechanical properties. However, few studies have focused on the evolution of the microstructure and mechanical properties of Mg–Gd–Y–Zr alloys during torsional deformation, and the related deformation mechanisms are rarely reported.

The aim of this work was to study the deformation mode, microstructure, and texture evolution of Mg–Gd–Y–Zr alloy during torsion at room temperature, and to evaluate the effect of the torsional process on the mechanical properties of the alloy.

## 2. Experimental Procedures

The material used in this study was extruded Mg–8Gd–3Y–0.4Zr (wt. %) alloy, which was obtained by semi-continuous casting, following homogenization and subsequent hot extrusion. The used material had a completely recrystallized structure with a basal texture and an average grain size of 15 μm, as has been reported in our previous work [20].The received materials were machined into dog-bone specimens with a gauge diameter of 8 mm and length of 18 mm, according to the American Society for Testing and Materials (ASTM) E8M-09 method, and were denoted as the as extruded (AE). Some AE samples were twisted at room temperature, with the twist axis parallel to the extrusion direction, and a twist rate of 2 rpm. The samples were twisted to 8%, 28%, and 51% shear strains and denoted as PT8, PT28, and PT51, respectively. The equivalent strain was estimated according to the following Equation (1):

(1) $$ɛ\;=\;\gamma /\sqrt{3}$$

where γ is the shear strain, which can be determined by Equation (2) [21]:

(2) γ = 2πNr/l

where N is the number of revolutions, *r* is the radial position of the sample, and l is the gauge length. For PT8, PT28, and PT51, the equivalent strain at the edge of the samples was determined to be 0.046, 0.162, and 0.294, respectively.

The microstructure characterization was conducted on the FEI Tecnai G2 F20 transmission electron microscope (TEM) (FEI Company, Hillsboro, OR, USA), operated at 200 kV, and texture determination was performed on the HELIOS NanoLab 600i Dual Beam Electron Microscope for EBSD ((FEI Company, Hillsboro, OR, USA) observation. Samples for EBSD were prepared by surface electrochemical polishing, and samples for TEM characterization were prepared by twin jet electropolishing. The polishing was conducted in a solution of 97% alcohol and 3% nitride acid at −35 °C. Following the ASTM procedure, the tensile tests were carried out on an Instron 3369 electronic universal machine with a strain rate of 10^−3^/s. All mechanical properties were taken from the arithmetic average of three parallel samples with a gauge diameter of 5 mm and a gauge length of 25 mm. The hardness tests were conducted on a Vickers hardness tester (Yanrun Company, Shanghai, China) with a load of 4.9 N and a dwelling time of 15 s. In order to ensure reliability, at least five indentations were made for each test and the average values were recorded.

## 3. Results and Discussion

### 3.1. Microstructure

**Figure 1:**
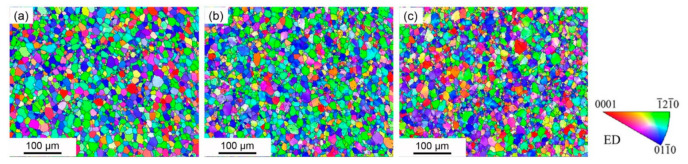
Inverse pole figures of the twisted samples in the edge region (**a**) PT8, (**b**) PT28, (**c**) PT51.

In order to investigate the texture variation and microstructure evolution, such as grain size and morphology, of the alloy after torsional deformation, EBSD analysis was conducted. Figure 1 presents the EBSD scans on the edge region of the twisted samples with different strains. When observed on the surface perpendicular to the twist axis, the size and shape of the grains did not change significantly, indicating that neither DRX nor grain fragmentation occurred. The inverse pole figures reveal that, compared with the starting material, the twisted samples have gradually reduced blue or green grains, and more purple or yellow grains, which indicates that the grains were gradually rotated with the alignment of the c-axis from the radius to the twist axis of the processed bars.

**Figure 2:**
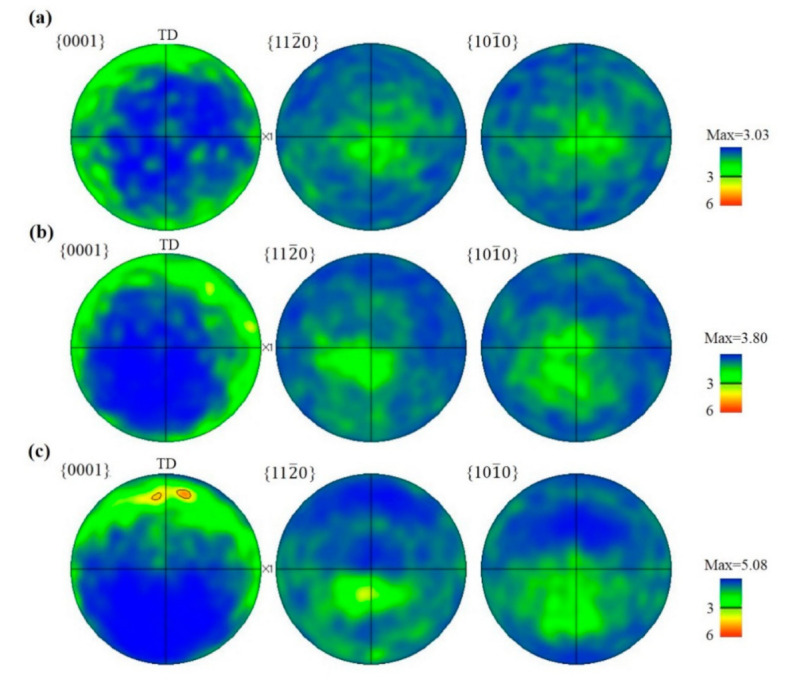
Pole figures corresponding to the $\left(0001\right)$, $\left(10\bar{1}0\right)$, and $\left(10\bar{1}1\right)$ planes of the twisted samples in the edge region (**a**) PT8, (**b**) PT28, (**c**) PT51.

The pole figures corresponding to $\left(0001\right)$, $\left(10\bar{1}0\right)$, $\left(10\bar{1}1\right)$ planes are shown in Figure 2. It can be seen that after the alloy was twisted, the basal pole was rotated from uneven distribution along the radius in the starting material, reaching a concentration slightly deviating from the radius to the twist axis, and the basal pole was located at a position about 20° from the twist axis in sample PT51. It is worth noting that after the gradual change, the $\left(10\bar{1}1\right)$ pole was “reset” on the twist axis. The shear deformation accommodated by <a> dislocations slipping can introduce Y fibers in the Mg alloy with the basal pole located at 60° away from the twist axis [22]. This texture with a basal pole located at about 70° away from the twist axis indicates that the activation of a large number of <a> dislocation slips and other deformation modes also have a considerable strain contribution.

**Figure 3:**
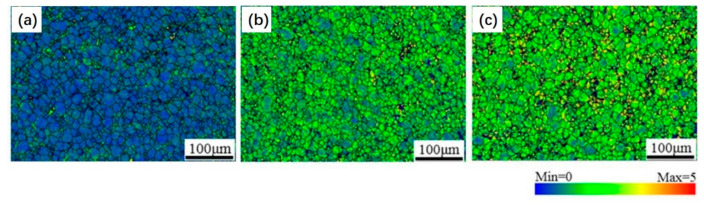
Local misorientation distributions of the twisted samples in the edge region (**a**) PT8, (**b**) PT28, (**c**) PT51.

In the twisted samples, especially in sample PT51, a strong variation in colors inside the grains was observed, indicating the occurrence of a change in orientation. In order to better present this feature, the corresponding local misorientation (LM) distribution is depicted in Figure 3. In all three samples, the LM was found to be higher near the grain boundaries and decreased toward the center of the grains, indicating an increased strain gradient from the center of the grains to the boundaries. This feature reveals that at the beginning of the twisting, the deformation was primarily accommodated in the local area near the grain boundaries until more areas inside the grains were trapped by the strain.

**Figure 4:**
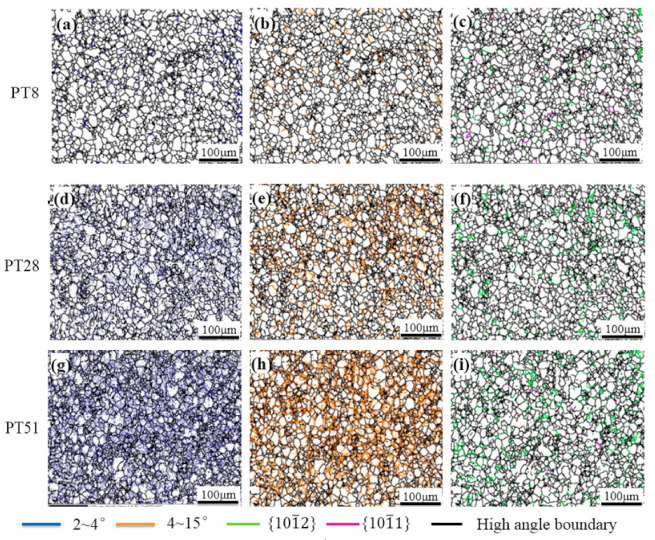
Boundary distributions of the twisted samples in the edge region (**a**–**c**) PT8, (**d**–**f**) PT28, (**g**–**i**) PT51. (**a**,**d**,**g**) for 2~4°, (**b**,**e**,**h**) for 4~15°and (**c**,**f**,**i**) for tension twin and compression twin.

The boundary angle distribution maps obtained from EBSD are shown in Figure 4, with green for the $\left(10\bar{1}2\right)$ twin boundary, pink for the $\left(10\bar{1}1\right)$ twin boundary, blue for the low angle boundaries (LABs) of 2–4 degrees, and orange for the LABs of 4–15 degrees. Unlike other deformations, such as forging or rolling conducted at room temperature, in this deformation, the alloys tend to be twinned easily, and very abundant twins were observed in the twisted alloy. Even in the sample whose equivalent strain became 0.294, the fraction of the $\left(10\bar{1}2\right)$ twin boundary was less than 3%, and the fraction of the $\left(10\bar{1}1\right)$ twinning boundary was less than 1%. Thus, it can be concluded that twinning is not a predominant mode in accommodating the torsional deformation. The fraction of the LABs with misorientation less than 15 degrees exceeded 7%.

**Figure 5:**
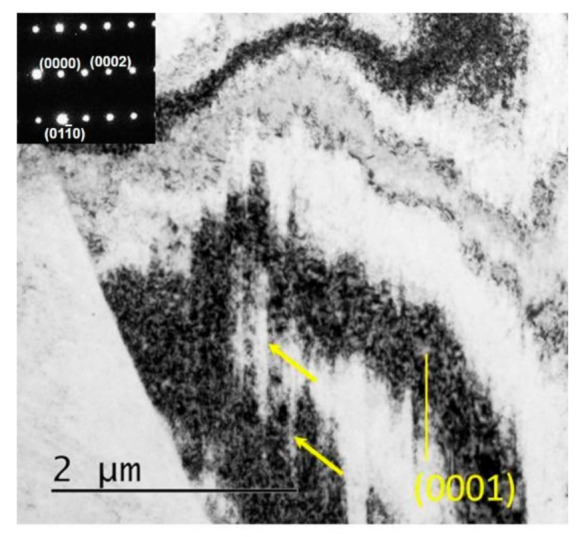
Transmission electron microscope image of sample PT51.

**Figure 6:**
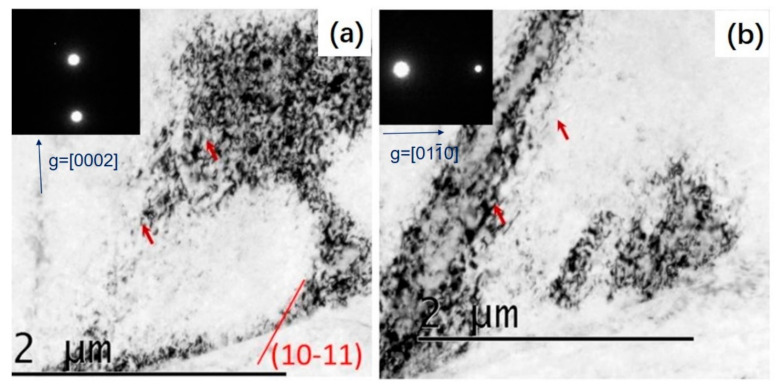
Transmission electron microscope images of sample PT51. (**a**) with a g vector of [0002], (**b**) with a g vector of $\left[01\bar{1}0\right]$.

In order to further understand the deformation mode and the formation mechanism of the LABs during torsion, TEM characterization was performed. As shown in the TEM bright field (BF) images in Figure 5, a large number of dislocation slipping traces were observed on the $\left(0001\right)$ plane, revealing the extensive activation of <a> dislocations. In addition to the basal slipping traces, dislocations or their projections on non-basal planes were also witnessed. As shown in Figure 6a, the BF image obtained with the electron beam parallel to $\left[10\bar{1}0\right]$ reveals a strong dislocation contrast. In order to determine the Burgers type of dislocations, the dislocation visibility criterion *g* × *b* ≠ 0 was used, and observations with two different g vectors were conducted, as shown in Figure 6b,c. It can be seen that the dislocations are both visible when observed with the g vector of $\left[0001\right]$ and the g vector of $\left[01\bar{1}0\right]$, indicating that the dislocations had both <c> and <a> components. As indicated by the red arrows, the most frequently observed dislocation lines are parallel to the $\left(10\bar{1}1\right)$ plane, demonstrating that they were <c+a> dislocations sliding on the $\left(10\bar{1}1\right)$ planes. The observed non-basal slipping traces were far fewer than the basal traces. Thus, it can be concluded that basal <a> dislocation slipping is the predominant mechanism, and <c+a> dislocation slipping on the $\left(10\bar{1}1\right)$ plane is subordinate in the simple shear deformation of Mg–Gd–Y–Zr alloy.

**Figure 7:**
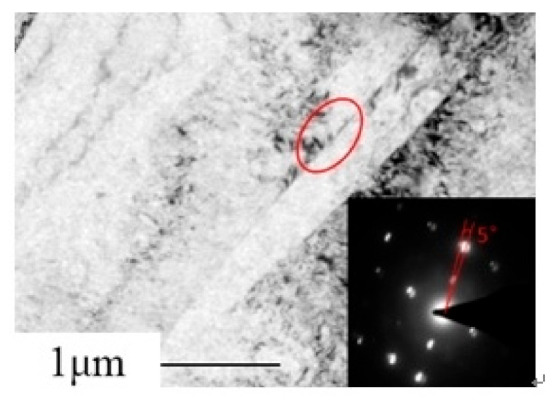
Transmission electron microscope image of sample PT51.

LABs of 2–15° have also been observed in the room-temperature forged counterpart, which were characterized to be boundaries between the micro-shear bands and the matrix [7]. The micro-shear bands were generated via piling up dislocations. The <a> dislocations on the basal plane dominated the present distortion and are considered to possess the highest sliding activity. Only after slipping into the prismatic planes were they observed to pile up or rearrange. Therefore, in the twisted alloy, almost no micro-shear bands were observed, as shown in Figure 7. LM is considered to be caused by severe local shear strain induced by the basal slippage near the grain boundaries, but there were no physical boundaries.

In addition, compared with cold-forged alloys, larger equivalent strains were imposed to the edge regions of the twisted alloy, and the twinning boundaries were much smaller, which indicates that twinning was restricted in simple shear deformation in Mg alloy.

### 3.2. Mechanical Properties

**Figure 8:**
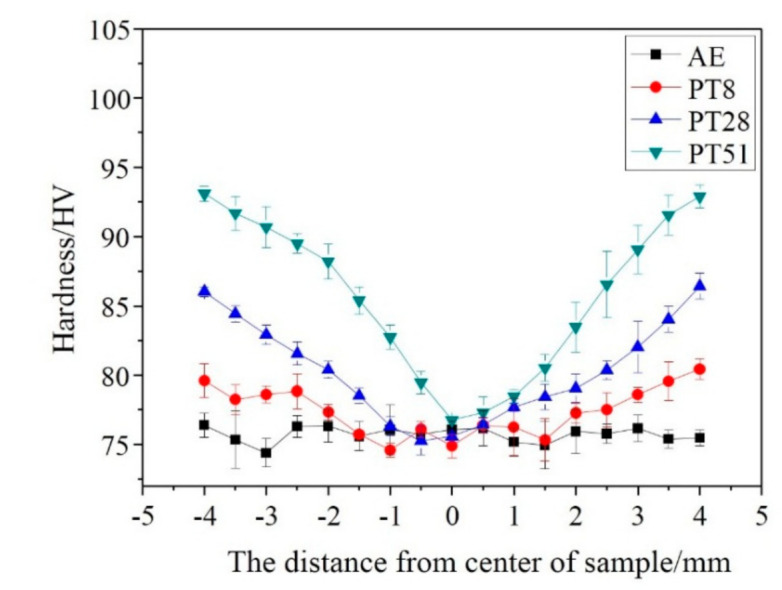
Vickers hardness variation along the radius of the twisted samples.

The mechanical properties of the alloy before and after torsion were investigated by micro-hardness measurements, and tensile and compression tests. Figure 8 shows the Vickers hardness variation along the radius of the alloy. After twisting, due to the gradient structure, the hardness of the sample gradually increased from the center to the edge. The edge region of the sample with the highest strain shows an increase from 76 HV to 93 HV.

**Figure 9:**
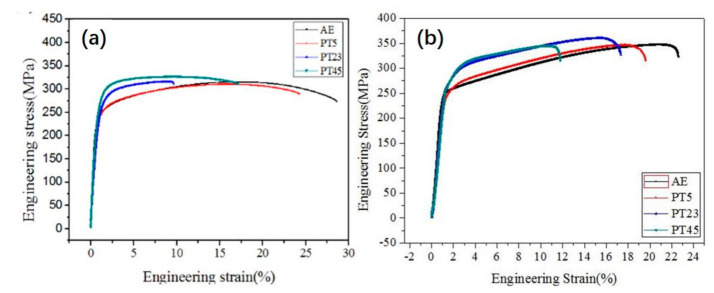
Stress–strain curves of the alloys in (**a**) tensile and (**b**) compression tests.

Figure 9 shows the stress–strain curves of the alloy whose tensile and compression directions were parallel to the twist direction and the corresponding properties are summarized in Table 1. With the increase of strain, the tensile and compressive strength gradually improved. The tensile yield strength increased from 218 MPa to 241 MPa, and the compressive strength increased from 239 MPa to 259 MPa.

Compared with the room-temperature forging [7], even a much higher equivalent strain was imposed on the sample, especially in the edge region, and the hardening and strengthening obtained by the room temperature torsion were decreased. This is attributed to the deformation mechanism and the resulting microstructure. The predominant activation of the basal <a> dislocations induced severe local shear and resulted in the strengthening of the dislocation forest in the alloy without producing boundary strengthening from the substructures, such as micro-shear bands and dislocation cells. The deformation-induced strengthening effect for the magnesium alloys depended, to a large extent, on the strain characteristics, not only because of the accumulated level but also because of a distinct difference in microstructures.

## 4. Conclusions

The torsional deformation of Mg–Gd–Y–Zr alloy was conducted at room temperature, and the related deformation mode, texture evolution, and mechanical properties were investigated. The deformation was predominantly accommodated by the basal <a> dislocations and subordinately by the <c+a> dislocations on the $\left(10\bar{1}1\right)$ planes, while the twinning was restricted. The deformation resulted in a modified Y fiber texture, the basal pole of which was slightly dispersed and rotated about 70° away from the twist axis. The distortion caused the tensile and compressive strength of the alloy to increase simultaneously.

## Figures and Tables

**Table 1:** Tensile mechanical properties of the samples.

Samples	Test	σ_y_ (MPa)	σ_u_ (MPa)	Δ (%)
AE	Ten	218	315	28.7
	Comp	239	349	21.8
PT8	Ten	221	310	24.5
	Comp	241	347	18.5
PT51	Ten	241	327	17.3
	Comp	259	346	11.0

## Data Availability

Data is contained within the article.
